# Generation of four-photon polarization entangled decoherence-free states with cross-Kerr nonlinearity

**DOI:** 10.1038/srep38233

**Published:** 2016-11-30

**Authors:** Meiyu Wang, Fengli Yan, Ting Gao

**Affiliations:** 1 College of Physics Science and Information Engineering, Hebei Normal University, Shijiazhuang 050024, China; 2 College of Mathematics and Information Science, Hebei Normal University, Shijiazhuang 050024, China

## Abstract

We propose a theoretical protocol for preparing four-photon polarization entangled decoherence-free states, which are immune to the collective noise. With the assistance of the cross-Kerr nonlinearities, a two-photon spatial entanglement gate, two controlled-NOT gates, a four-photon polarization entanglement gate are inserted into the circuit, where X homodyne measurements are aptly applied. Combined with some swap gates and simple linear optical elements, four-photon polarization entangled decoherence-free states which can be utilized to represent two logical qubits, |0〉_*L*_ and |1〉_*L*_ are achieved at the output ports of the circuit. This generation scheme may be implemented with current experimental techniques.

Entanglement^1^,^2^,^3^ plays an important role in quantum information processing, mainly including quantum computation^4^ and quantum communication. It is the information carrier in some interesting branches of quantum communication, such as quantum key distribution^5^, quantum secret sharing^6^,^7^,^8^, quantum secure direct communication^9^,^10^,^11^, teleportation^12^, quantum dense coding^13^,^14^, and so on. Most of the above applications require maximally entangled states or noiseless quantum channels. However, in a realistic situation, decoherence, induced by uncontrolled coupling between a quantum system and the environment, is inevitable. When qubits are coupled to the environment, the quantum superposition and coherence are easily destructed, and as a result the maximally entangled state collapses into a non-maximally entangled one or even a mixed state. This will degrade the fidelity and security of quantum communication. To overcome this flaw, some specific entangled states, which are called decoherence-free states^15^,^16^,^17^, are proposed. Decoherence-free states, no matter how strong the qubit-environment interaction, exhibit some symmetry, so the quantum states are invariant under this interaction. Therefore, the decoherence-free states are very useful for long-distance quantum information transmission and storage.

Due to the fact that photons have the merits of higher speed, lower decoherence, easier manipulation, and lower energy cost compared with more massive qubits, polarization photons are destined to have a central role in long-distance communication. Recently, the encoding in decoherence-free states of polarization photons to overcome collective decoherence attracts the extensively attention. An optical experiment has been reported to overcome collective noise by encoding quantum information into the decoherence-free state^18^. For two qubits, there is only one decoherence-free singlet state, i.e., 
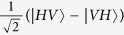
, where *H* and *V* denote horizontal and vertical linear polarizations respectively. Therefore, it is not sufficient to fully protect the quantum information of an arbitrary logical qubit against collective noise. Another nontrivial example is the four-photon polarization entangled decoherence-free state


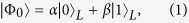


with


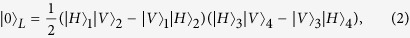



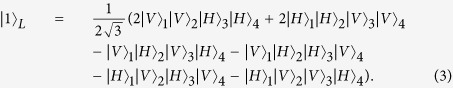


The dimension of the above four-qubit decoherence-free state in Eq. (1) is 2, and thus it is sufficient to fully protect an arbitrary logical qubit against collective noise in contrast to the two-qubit state. With its interesting applications, Bourennane *et al*.^19^ have generated four-photon polarization entangled decoherence-free states via a spontaneous parametric down-conversion source. Recently, Zou *et al*.^20^ and Gong *et al*.^21^ proposed two different schemes to generate four-photon polarization entangled decoherence-free states based on linear optical elements and postselection strategy. Subsequently, Xia *et al*.^22^ presented a protocol for the controlled generation of the four-photon polarization entangled decoherence-free state with conventional photon detectors. In 2010, Wang *et al*.^23^ proposed a probabilistic linear-optics-based scheme for local conversion of four Einstein-Podolsky-Rosen photon pairs into four-photon polarization entangled decoherence-free states. In 2013, Xia *et al*.^24^ also put forward a probabilistic protocol for preparation of four-photon polarization entangled decoherence-free states with the help of the cross-Kerr nonlinearity medium.

In this paper, we present an alternative scheme to generate the four-photon polarization entangled decoherence-free states with the assistance of the cross-Kerr nonlinearities. The states representing the logical qubits |0〉_*L*_ and |1〉_*L*_ can be achieved at different output ports of two beam splitters, combined with the output ports of the photon 3 and the photon 4. The rest of the paper is organized as follows. In Sec. II, we show how to generate these two logical qubits in the four-photon polarization entangled decoherence-free states based on the weak cross-Kerr nonlinearities. The discussion and conclusion are presented in Sec. III.

## Generations of four-qubit entangled decoherence-free states

For the sake of the clearness, let us first introduce the cross-Kerr nonlinearity, which was first used by Chuang and Yamamoto to realize the simple optical quantum computation^25^. The interaction Hamiltonian has the form 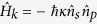, where 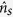 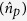 is the photon-number operators of the signal (probe) mode, and *κ* is the strength of the nonlinearity. If the signal field contains *n* photons and the probe field is in an initial coherent state with amplitude *α*, the cross-Kerr nonlinearity interaction causes the combined signal-probe system to evolve as follows:


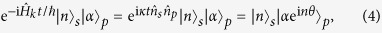


where *θ* = *κt* with *t* being the interaction time. It is easy to observe that the Fock state is unaffected by the interaction but the coherent state picks up a phase shift *nθ* directly proportional to the number of photons *n* in the signal mode. One can exactly obtain the information of photons in the Fock state but not destroy them by detecting the probe mode with a general homodyne-heterodyne measurement. The cross-Kerr nonlinearity between photons offers an ideal playground for quantum state engineering, and a number of applications have been studied, such as constructing nondestructive quantum nondemoliton detectors (QND)^26^,^27^, deterministic entanglement distillation^28^, logic-qubit entanglement^29^,^30^, generation of multi-photon entangled states and decoherence-free states^24^,^31^,^32^,^33^,^34^,^35^,^36^.

**Figure 1:**
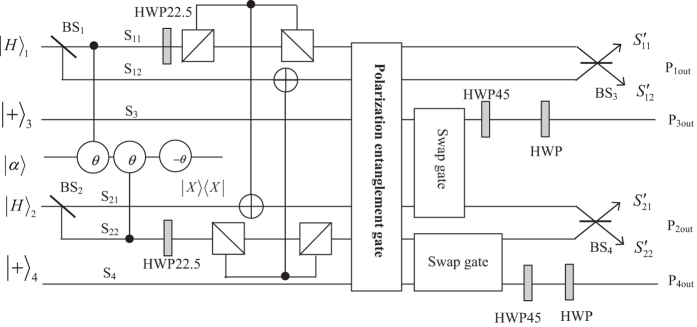
Illustration plot for generating four-photon polarization entangled decoherence-free state with the help of the cross-Kerr nonlinearities. The symbol BS denotes the beam splitter which has equal probability (50:50) of transmission and reflection. HWP22.5, HWP45, and HWP denote half-wave plates which realize the Hadamard transformation operation, single photon *σ*_*x*_ operation, and single photon *σ*_*z*_ operation respectively. In the construction of the circuit, after two Controlled-Not gates, a polarization entanglement gate and two swap gates need to be performed, which can be seen in Figs 2 and 3 respectively. Before four photons leave the circuit, the four potential paths of the photon (1, 2) are coherently combined by *BS*_3_ and *BS*_4_ for obtaining the different four-photon polarization entanglement decoherence-free states.

In what follows, we explain the detailed procedures for generating the four-photon polarization entangled docoherence-free states abided by the following processes, which is also illustrated in Fig. 1.

Assume the four single photons are initially prepared in the state 
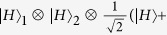

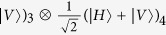
, and let them enter into the circuit shown in Fig. 1 from the input ports. The first step is to create the spatial entanglement of the photons 1 and 2. Passing through beam splitters, BS_1_ and BS_2_, which have the following function between two input modes (a,b) and two output modes (c,d): 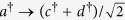, 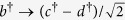, the photons (1, 2) enter into the paths (*S*_11_, *S*_12_) and the paths (*S*_21_, *S*_22_) respectively. Accompanying with the coherent state, the photons (1, 2) enter into Kerr media. Then, the state of photons (1, 2) with the coherent state |*α*〉 evolves as


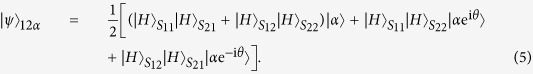


Performing an X homodyne measurement on the coherent state with *α* real, there are two measurement outcomes corresponding to scenarios of phase shift (0, ±*θ*). For the convenience of analysis, we expand the state in terms of the eigenstates of the X operator:


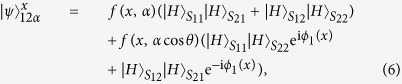


where the coefficients^37^ are


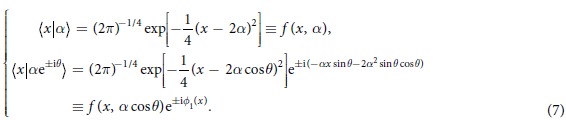


Explicitly, if zero phase shift occurs, the spatial entangled state of the photons (1, 2) is created and can be written as


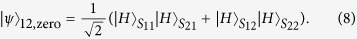


Otherwise, another measurement outcome (nonzero phase shift) is obtained, a phase shift operation 2*ϕ*_1_(*x*) should be performed on the photon 2 passing through the path *S*_21_ to erase the phase difference between two terms of 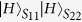 and 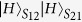. By omitting a global phase *ϕ*_1_(*x*), the photons (1, 2) are in the following spatial entangled state


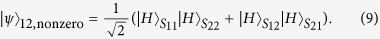


Without considering other conditions, there is a small probability of error to distinguish the state in Eq. (8) and the state in Eq. (9) from each other due to the overlap of the measurement functions *f(x, α*) and *f(x, α* cos *θ*), which is given by 
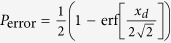
, *x*_*d*_ = 2*α*(1 − cos *θ*). It is less than 10^−5^ when the distance *x*_*d*_ ~ *αθ*^2^ > 9^26^.

For simplifying description in the later processes, we take an example as the representative of two different scenarios of phase shift. If zero phase shift is witnessed by the X homodyne measurement, half wave plates, HWP22.5°s, are inserted into the paths *S*_11_, *S*_22_ at first, which function as Hadamard transformation operations to transform the state of the photons (1, 2) from 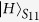 and 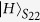 to 
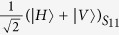
 and 
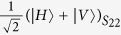
. Then two controlled-NOT (CNOT) gates are performed on two paths (*S*_11_, *S*_21_) (photon 1 as control photon and photon 2 as target photon), and paths (*S*_22_, *S*_12_) (photon 2 as control photon and photon 1 as target photon) respectively. The CNOT gate is important in the experimental realization. Knill *et al*.^38^ firstly proposed a probabilistic CNOT gate on two photonic qubits by using linear optical elements and postselection. The cross-Kerr nonlinearity has also been used to implement the CNOT gate^26^,^39^,^40^. After two CNOT gates, the four-photon system will evolve into


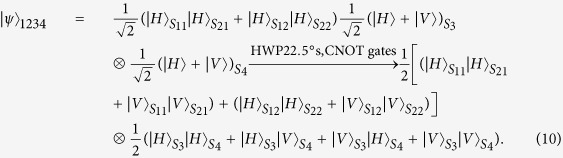


**Figure 2:**
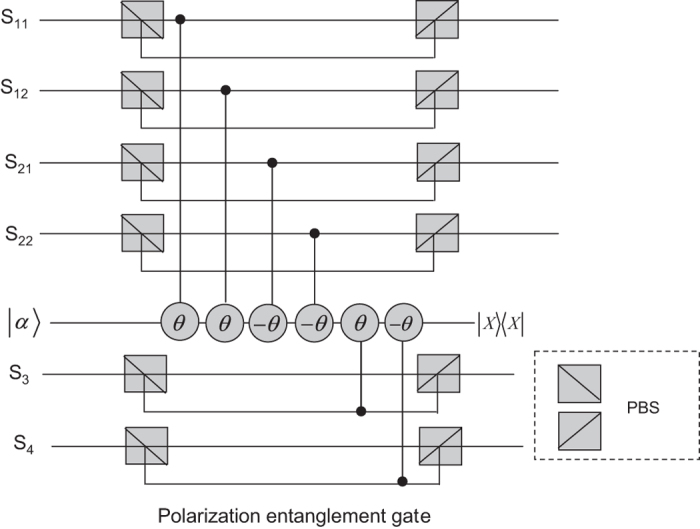
Illustration plot for depicting a polarization entanglement gate. The polarization beam splitters (PBS) reflect the vertical polarization |*V*〉 mode and transmit the horizontal polarization |*H*〉 mode. Influenced by cross-Kerr nonlinearities, the photon 1 in the horizontal polarization state and the photon 3 in the vertical polarization state enable the coherent state |*α*〉 to pick up the phase shift *θ*, while the photon 2 in the horizontal polarization state and the photon 4 in the vertical polarization state enable the coherent state |*α*〉 to pick up the phase shift −*θ*.

A polarization entanglement gate illustrated in Fig. 2 is put into the paths (*S*_11_, *S*_12_, *S*_21_, *S*_22_, *S*_3_, *S*_4_) of the photons (1, 2, 3, 4) to entangle them with the polarization degree of freedom. Affected by cross-Kerr nonlinearities, the horizontal polarization mode of photons (1, 2) via the paths *S*_11_, *S*_12_, *S*_21_, *S*_22_ will accumulate the phase shift *θ*, −*θ* respectively while the vertical polarization mode of photons (3, 4) will accumulate the phase shift *θ*, −*θ* respectively on the coherent state |*α*〉. As the consequence of the nonlinear interaction between photons and the coherent state, the state of the whole system can be expressed as


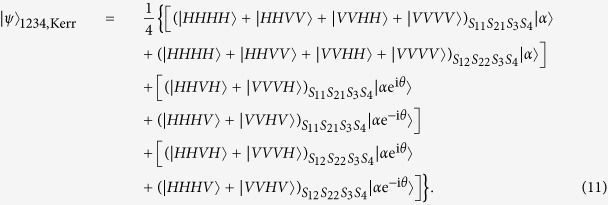


After the photons leave Kerr media, the X homodyne measurement is performed on the coherent state. If zero phase shift occurs, no phase modulation is necessary. Otherwise, if nonzero phase shift of the coherent state presents on the measurement setup, a phase shift 2*ϕ(x*) operation should be performed on the photon 3 in the path *S*_31_. Moreover, a HWP45° should be inserted into the path *S*_3_ to perform *σ*_*x*_ operation on the photon 3. So the four-photon state can be denoted as


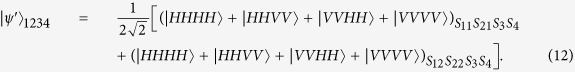


Here, two swap gates need to be performed on two photons in the paths (*S*_21_, *S*_3_) and (*S*_22_, *S*_4_) respectively to swap them. A swap gate is an important two-qubit logic gate. In terms of the basis of {|00〉, |01〉, |10〉, |11〉}, the swap gate can be represented as the following matrix:


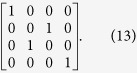


**Figure 3:**
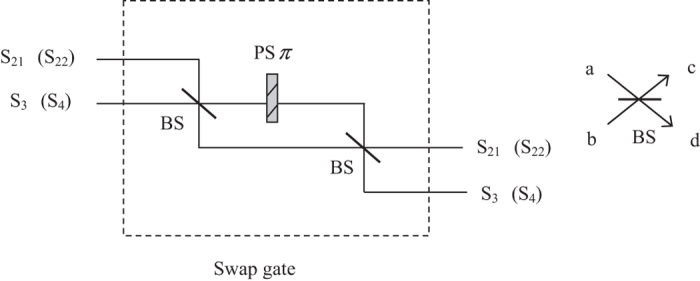
Illustration plot for depicting the swap gate. The symbol PS *π* denotes the phase shift *π* executed on the photon passing through the line it is inserted. A beam splitter has the following function between two input modes (a,b) and two output modes (c,d): 
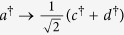
, 
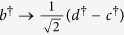
.

In practice, the swap gate transformation can be yielded by the Hong-Ou-Mandel interference^41^ in the Mach-Zehnder interferometer^39^,^42^, illustrated in Fig. 3. Two beam splitters constitute a Mach-Zehnder interferometer. Additionally, the phase shifter PS *π* denotes the phase shift *π* executed on the photon passing through the line it is inserted.

After two swap gate operations, the state denoted in Eq. (12) is changed to


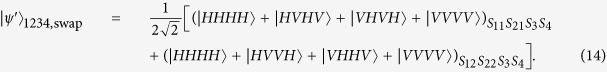


Then, a local unitary operation *σ*_*y*_ should be performed on photon 3 and 4, respectively, which can be realized by the combination of a HWP45° and a HWP. So the above state can be denoted as


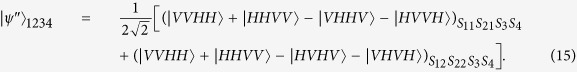


Due to the presence of BS_3_ and BS_4_, the photons (1, 2) leave the paths (*S*_11_, *S*_12_) and the paths (*S*_21_, *S*_22_) to the paths 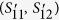 and the paths 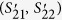 according to the following rules, 
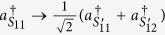
, 
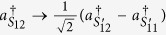
, 
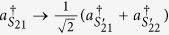
, 
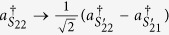
. Correspondingly, at the output ports, the state of four photons expressed as Eq. (15) is transformed to


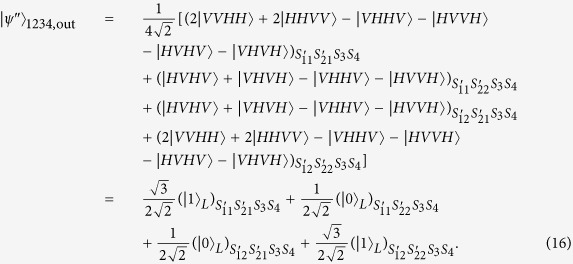


From the above equation, we can see that by detecting the outputs of the four photons, the logical qubit |0〉_*L*_ can be obtained with the total prabability of 25% at the output ports of 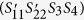 or 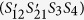. As for the logical qubit |1〉_*L*_, it can be obtained with the total prabability of 75% at the output ports of 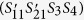 or 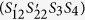.

As for another scenario, if we obtain the spatial entangled state denoted as Eq. (9), with a similar process, we can also obtain the four-photon polarization entangled decoherence-free states. It is worth noting that the state denoted as Eq. (9) is the same as Eq. (8) when a swap gate is inserted into the path *S*_21_ and *S*_22_, so far, the preparation of four-photon polarization entangled decoherence-free states if fullfilled.

## Discussion and Conclusion

We now give a brief discussion about the experimental feasibility of protocol with the current experimental technology. First of all, in the input ports, single-photon resources are used. The complete technology of these single photons is yet to be established^43^,^44^,^45^,^46^. Currently single-photon sources in signal modes can be achieved from the collinear type II spontaneous parametric down conversion^47^. As down conversion experiments are intrinsically probabilistic due to the statistical creation property of the photon pairs, the scheme will be in a sense probabilistic too in view of the usage of single-photon sources. Thus, more efficient ones are demanded for our setup. Second, in the present scheme, two CNOT gates are performed, which can be realized following the refs 26,38,39,40. However, these methods are at the best, nearly deterministic, so our scheme could be nearly deterministic. Third, in our protocol, we exploit the cross-Kerr nonlinearities medium in the spatial entanglement process and performing the polarization entanglement gate. It should be noted that in actual experiments, many factors will affect the perfect performance of cross-Kerr nonlinearities, such as dispersion, self-phase modulation, molecular vibrations in Kerr media, etc. Shapiro *et al*.^48^ analyzed the cross-Kerr nonlinear interaction and showed that single-mode cross-Kerr nonlinearities is not available for quantum information processing. Recently, Gea-Banacloche^49^ pointed out that the large phase shifts via the giant Kerr effect with single-photon wave packets is impossible at present. A proper physics systems providing larger strength of cross-Kerr nonlinearity should be atomic ensemble, and the fundamental problem with the cross-Kerr nonlinearity in atomic ensemble was discussed by Gea-Banacloche^49^, and He and Scherer^50^. Finally, the experiment feasibility of the present protocols also depends on the veracity of the X homodyne measurement. For the X homodyne measurement, we only consider the error chiefly coming from the overlap adjacent curves because of the fact that the coherent states of the probe beam with different phase shifts are not completely orthogonal. In fact, it is only one type of detection error in homodyne, other errors, such as the noises in detection, the reduced fidelity to the process in Eq. (4) due to multi-mode effect and decoherence, etc., also exist in a realistic implementation. Exploiting the appropriate measurement methods, the disadvantageous influence can be overcome or alleviated and the error probability will be decreased. In 2010, Wittmann *et al*.^51^ investigated quantum measurement strategies capable of discriminating two coherent states using a homodyne detector and a photon number resolving (PNR) detector. In order to lower the error probability, the postselection strategy is applied to the measurement data of homodyne detector as well as a PNR detector. They indicated that the performance of the new displacement controlled PNR is better than homodyne receiver.

To summarize, we have proposed a theoretical protocol for preparing four-photon polarization entangled decoherence-free states with the assistance of the cross-Kerr nonlinearity. In our protocol, combined with some swap gates and simple linear optical elements, a two-photon spatial entanglement gate, two CNOT gates, a four-photon polarization entanglement gate are applied. We hope our work will afford facilities for other practical implementations of quantum information processing based on optics.

## Additional Information

**How to cite this article**: Wang, M. *et al*. Generation of four-photon polarization entangled decoherence-free states with cross-Kerr nonlinearity. *Sci. Rep.* **6**, 38233; doi: 10.1038/srep38233 (2016).

**Publisher’s note:** Springer Nature remains neutral with regard to jurisdictional claims in published maps and institutional affiliations.

## References

[b1] Horodecki R., Horodecki P., Horodecki M. & Horodecki K. Quantum entanglement. Rev. Mod. Phys. 81, 865–942 (2009).

[b2] Yan F. L., Gao T. & Chitambar E. Two local observables are sufficient to characterize maximally entangled states of *N* qubits. Phys. Rev. A 83, 022319 (2011).

[b3] Gao T., Yan F. L. & van Enk S. J. Permutationally invariant part of a density matrix and nonseparability of *N*-qubit states. Phys. Rev. Lett. 112, 180501 (2014). 24856681 10.1103/PhysRevLett.112.180501

[b4] DiVincenzo D. P. Quantum gates and circuits. Proc. R. Soc. London A 454, 261–276 (1998).

[b5] Ekert A. K. Quantum cryptography based on Bell’s theorem. Phys. Rev. Lett. 67, 661–663 (1991). 10044956 10.1103/PhysRevLett.67.661

[b6] Hillery M., Bužek V. & Berthiaume A. Quantum secret sharing. Phys. Rev. A 59, 1829–1834 (1999).

[b7] Deng F. G. . Multiparty quantum-state sharing of an arbitrary two-particle state with Einstein-Podolsky-Rosen pairs. Phys. Rev. A 72, 044301 (2005).

[b8] Yan F. L. & Gao T. Quantum secret sharing between multiparty and multiparty without entanglement. Phys. Rev. A 72, 012304 (2005).

[b9] Deng F. G., Long G. L. & Liu X. S. Two-step quantum direct communication protocol using the Einstein-Podolsky-Rosen pair block. Phys. Rev. A 68, 042317 (2003).

[b10] Gao T., Yan F. L. & Wang Z. X. Deterministic secure direct communication using GHZ states and swapping quantum entanglement. J. Phys. A 38, 5761–5770 (2005).

[b11] Zhu A. D., Xia Y., Fan Q. B. & Zhang S. Secure direct communication based on secret transmitting order of particles. Phys. Rev. A 73, 022338 (2006).

[b12] Bennett C. H. . Teleporting an unknown quantum state via dual classical and Einstein-Podolsky-Rosen channels. Phys. Rev. Lett. 70, 1895–1899 (1993). 10053414 10.1103/PhysRevLett.70.1895

[b13] Bennett C. H. & Wiesner S. J. Communication via one- and two-particle operators on Einstein-Podolsky-Rosen states. Phys. Rev. Lett. 69, 2881–2884 (1992). 10046665 10.1103/PhysRevLett.69.2881

[b14] Liu X. S., Long G. L., Tong D. M. & Feng L. General scheme for superdense coding between multiparties. Phys. Rev. A 65, 022304 (2002).

[b15] Duan L. M. & Guo G. C. Preserving coherence in quantum computation by pairing quantum bits. Phys. Rev. Lett. 79, 1953–1956 (1997).

[b16] Zanardi P. & Rasetti M. Noiseless quantum codes. Phys. Rev. Lett. 79, 3306–3309 (1997).

[b17] Kempe J., Bacon D., Lidar D. A. & Whaley K. B. Theory of decoherence-free fault-tolerant universal quantum computation. Phys. Rev. A 63, 042307 (2001). 10.1103/PhysRevLett.85.1758 10970607

[b18] Altepeter J. B. . Experimental investigation of a two-qubit decoherence-free subspace. Phys. Rev. Lett. 92, 147901 (2004). 15089574 10.1103/PhysRevLett.92.147901

[b19] Bourennane M. . Decoherence-free quantum information processing with four-photon entangled states. Phys. Rev. Lett. 92, 107901 (2004). 15089244 10.1103/PhysRevLett.92.107901

[b20] Zou X. B., Shu J. & Guo G. C. Simple scheme for generating four-photon polarization-entangled decoherence-free states using spontaneous parametric down-conversions. Phys. Rev. A 73, 054301 (2006).

[b21] Gong Y. X. . Generation of arbitrary four-photon polarization-entangled decoherence-free states. Phys. Rev. A 77, 042317 (2008).

[b22] Xia Y., Song J., Song H. S. & Zhang S. Controlled generation of four-photon polarization-entangled decoherence-free states with conventional photon detectors. J. Opt. Soc. Am. B 26, 129–132 (2009).

[b23] Wang H. F. . Local conversion of four Einstein-Podolsky-Rosen photon pairs into four-photon polarization-entangled decoherence-free states with non-photon-number-resolving detectors. Opt. Exp. 19, 25433–25440 (2011). 10.1364/OE.19.025433 22273935

[b24] Xia Y. . Effective protocol for preparation of four-photon polarization-entangled decoherence-free states with cross-Kerr nonlinearity. J. Opt. Soc. Am. B 30, 421–428 (2013).

[b25] Chuang I. L. & Yamamoto Y. Simple quantum computer. Phys. Rev. A 52, 3489–3496 (1995). 9912648 10.1103/physreva.52.3489

[b26] Nemoto K. & Munro W. J. Nearly deterministic linear optical controlled-NOT gate. Phys. Rev. Lett. 93, 250502 (2004). 15697884 10.1103/PhysRevLett.93.250502

[b27] Barrett S. D. . Symmetry analyzer for nondestructive Bell-state detection using weak nonlinearities. Phys. Rev. A 71, 060302 (2005).

[b28] Sheng Y. B. & Zhou L. Deterministic entanglement distillation for secure double-server blind quantum computation. Sci. Rep. 5, 7815 (2015). 25588565 10.1038/srep07815 PMC4295105

[b29] Ding D., Yan F. L. & Gao T. Preparation of *km*-photon concatenated Greenberger-Horne-Zeilinger states for observing distinctive quantum effects at macroscopic scales. J. Opt. Soc. Am. B 30, 3075–3078 (2013).

[b30] Sheng Y. B. & Zhou L. Two-step complete polarization logic Bell-state analysis. Sci. Rep. 5, 13453 (2015). 26307327 10.1038/srep13453 PMC4549687

[b31] He Y. Q., Ding D., Yan F. L. & Gao T. Exploration of multiphoton entangled states by using weak nonlinearities. Sci. Rep. 6, 19116 (2016). 26751044 10.1038/srep19116 PMC4707534

[b32] Ding D., Yan F. L. & Gao T. Entangler and analyzer for multiphoton Greenberger-Horne-Zeilinger states using weak nonlinearities. Sci. Chin. Phys. Mech. Astron. 57, 2098–2103 (2014).

[b33] He Y. Q., Ding D., Yan F. L. & Gao T. Exploration of photon-number entangled states using weak nonlinearities. Opt. Exp. 23, 21671 (2015). 10.1364/OE.23.021671 26368146

[b34] Dong L. . Nearly deterministic preparation of the perfect W state with weak cross-Kerr nonlinearities. Phys. Rev. A 93, 012308 (2016).

[b35] Lin Q. & He B. Highly efficient processing of multi-photon states. Sci. Rep. 5, 12792 (2015). 26245489 10.1038/srep12792 PMC4526856

[b36] Dong Li. . Generation of three-photon polarization-entangled decoherence-free states. Ann. Phys. 371, 287–295 (2016).

[b37] Gardiner C. W. & Zoller P. Quantum Noise, p. 113 (Springer, Berlin, 2000).

[b38] Knill E., Laflamme R. & Milburn G. J. A scheme for efficient quantum computation with linear optics. Nature 409, 46–52 (2001). 11343107 10.1038/35051009

[b39] Lin Q. & Li J. Quantum control gates with weak cross-Kerr nonlinearity. Phys. Rev. A 79, 022301 (2009).

[b40] Beenakker C. W. J., DiVincenzo D. P., Emary C. & Kindermann M. Charge detection enables free-electron quantum computation. Phys. Rev. Lett. 93, 020501 (2004). 15323887 10.1103/PhysRevLett.93.020501

[b41] Hong C. K., Ou Z. Y. & Mandel L. Measurement of subpicosecond time intervals between two photons by interference. Phys. Rev. Lett. 59, 2044–2046 (1987). 10035403 10.1103/PhysRevLett.59.2044

[b42] Milburn G. J. Quantum optical fredkin gate. Phys. Rev. Lett. 62, 2124–2127 (1989). 10039862 10.1103/PhysRevLett.62.2124

[b43] Brunel C., Lounis B., Tamarat P. & Orrit M. Triggered source of single photons based on controlled single molecule fluorescence. Phys. Rev. Lett. 83, 2722–2725 (1999).

[b44] Michler P. . A quantum dot single-photon turnstile device. Science 290, 2282–2285 (2000). 11125136 10.1126/science.290.5500.2282

[b45] Santori C. . Triggered single photons from a quantum dot. Phys. Rev. Lett. 86, 1502–1505 (2001). 11290178 10.1103/PhysRevLett.86.1502

[b46] Wang S. M., Ma H. Q. & Wu L. A. A single photon source based on entangled photon pairs. Acta Phys. Sin. 58, 717–721 (2009).

[b47] Su S. L., Cheng L. Y., Wang H. F. & Zhang S. An economic and feasible scheme to generate the four-photon entangled state via weak cross-Kerr nonlinearity. Opt. Commun. 293, 172–176 (2013).

[b48] Shapiro J. H. & Razavi M. Continuous-time cross-phase modulation and quantum computation. New J. Phys. 9, 16 (2007).

[b49] Gea-Banacloche J. Impossibility of large phase shifts via the giant Kerr effect with single-photon wave packets. Phys. Rev. A 81, 043823 (2010).

[b50] He B. & Scherer A. Continuous-mode effects and photon-photon phase gate performance. Phys. Rev. A 85, 033814 (2012).

[b51] Wittmann C., Andersen U. L., Takeoka M. & Leuchs G. Discrimination of binary coherent states using a homodyne detector and a photon number resolving detector. Phys. Rev. A 81, 062338 (2010). 10.1103/PhysRevLett.104.100505 20366409

